# Novel ADCs and combination therapy in urothelial carcinoma: latest updates from the 2023 ASCO-GU Cancers Symposium

**DOI:** 10.1186/s13045-023-01475-9

**Published:** 2023-07-28

**Authors:** Jiazheng Yu, Siyu Wu, Rong Li, Yuanhong Jiang, Jianyi Zheng, Zeyu Li, Mingyang Li, Kerong Xin, Xiaojiao Guan, Shijie Li, Xiaonan Chen

**Affiliations:** 1 Department of Urology, Shengjing Hospital of China Medical University, Shenyang, 110004 Liaoning People’s Republic of China; 2 Department of Pathology, Shengjing Hospital of China Medical University, Shenyang, 110004 Liaoning People’s Republic of China

**Keywords:** ADCs, Enfortumab vedotin, Sacituzumab govitecan, Disitamab vedotin, Urothelial carcinoma

## Abstract

Antibody–drug conjugates (ADCs) combine the cytotoxicity of small-molecule drugs with antibody targeting. Due to their precise and powerful effect, they have become a new hotspot and an important trend in the research and development of anti-tumor antibody drugs. Every year, exciting new developments and innovations in the treatment of urological tumors are introduced at the American Society of Clinical Oncology-Genitourinary (ASCO-GU) Cancers Symposium. In this article, we summarize some of the most impressive advances in new clinical trials and clinical data on ADCs in the 2023 ASCO-GU Cancers Symposium for the treatment of urothelial carcinoma.

**To the editor:**

Each year, exciting developments in urological tumors are introduced at the American Society of Clinical Oncology-Genitourinary (ASCO-GU) Cancers Symposium. In this article, we review the impressive progress made in new clinical trials and data concerning antibody–drug conjugates (ADCs) for urothelial carcinoma treatment from the 2023 Symposium.

## Enfortumab vedotin in urothelial carcinoma

Enfortumab vedotin (EV) is an ADCs formed by joining a humanized Nectin-4 targeted IgG1 monoclonal antibody, enfortumab, and a microtubule-disrupting agent, monomethyl auristatin E (MMAE), through a cleavable mc-val-cit-PABC linker. The EV-103 cohort K (NCT03288545) evaluated EV or EV + Pembrolizumab (Pembro) as a first-line therapy for cisplatin-ineligible patients with locally advanced or metastatic urothelial cancer (la/mUC). Patients were randomized 1:1 to receive EV monotherapy on days 1 and 8, or in combination with Pembro on day 1 of the 3-week cycles. EV monotherapy showed an objective response rate (ORR) of 45.2% (95% CI 33.5–57.3), while the EV + Pembro combination demonstrated an ORR of 64.5% (95% CI 52.7–75.1). Treatment-related adverse events (TRAEs) in the EV + Pembro arm included skin reactions (67.1%) and peripheral neuropathy (60.5%). TRAEs were observed in 68.4% of the patients. This led to the interruption of EV or Pembro, with 48.7% of patients requiring EV dose reduction [1]. This established the foundation for accelerated approval of EV + Pembro by the US Food and Drug Administration (FDA), for cisplatin-ineligible mUC in April 2023.

Another ongoing phase 1 trial (NCT05014139) is studying intravesical EV infusion in high-risk, Bacillus Calmette-Guérin-unresponsive patients with non-muscle-invasive bladder cancer [2].

## Sacituzumab govitecan in urothelial carcinoma

Sacituzumab govitecan (SG) is an ADC composed of an anti-Trop-2 antibody, sacituzumab, and a topoisomerase I inhibitor, SN-38, bound through the hydrolyzable linker CL2A. The ongoing phase 2 trial TROPHY-U-01, evaluated SG monotherapy and combination therapy in patients with la/mUC (NCT03547973). Cohort 1 demonstrated a 28% ORR (95% CI 20.2–37.6) in 113 patients with la/mUC, who had progressed after platinum-based chemotherapy and checkpoint inhibitor (CPI) treatment. Median overall survival (med-OS) was 10.9 months (95% CI 8.9–13.8), median progression-free survival (med-PFS) was 5.4 months (95% CI 3.5–6.9), and median duration of response (med-DOR) was 6.1 months (95% CI 4.7–9.7, *n* = 32), leading to accelerated FDA approval for patients in cohort 1 [3]. Cohort 2 assessed SG monotherapy in patients with platinum-ineligible mUC who showed disease progression after CPI treatment [4]. Cohort 3 assessed combined SG and Pembro treatment in 41 patients with mUC, after platinum-based therapy, which supported the need for further evaluation of SG and CPI combination treatment in patients with mUC [5]. The common TRAEs in the cohort included febrile and non-febrile neutropenia, anemia, leukopenia, fatigue, and diarrhea. Anemia and fatigue appeared to be more SG-related, whereas diarrhea was more CPI-related. Cohort 5 evaluated SG + zimberelimab (ZIM) versus ZIM alone versus avelumab for switch maintenance in patients with mUC who received gemcitabine (GEM)/cisplatin without progressive disease [6]. In Cohort 6, we assessed SG monotherapy versus SG + CPI combinations (SG + ZIM, SG + ZIM + domvanalimab) versus carboplatin/GEM, followed by avelumab maintenance, in treatment-naive cisplatin-ineligible patients with la/mUC [7].

Another ongoing trial (NCT04863885) is investigating ipilimumab plus nivolumab combined with SG in cisplatin-ineligible patients with mUC. Phase 1 results: ORR was 66.6% in 6 patients, med-DOR was 9.2 months (95% CI 4.6–12.0), and med-PFS was 8.8 months (95% CI 3.8–NR). The TRAEs included anemia, neutropenia, pruritus, fatigue, diarrhea, lymphopenia, and arthralgia. A phase 2 trial with biomarker analysis is ongoing [8].

## Disitamab vedotin in urothelial carcinoma

Disitamab vedotin (DV; RC48) is an ADC composed of a human epidermal growth factor receptor 2 (HER2)-targeted monoclonal antibody, hertuzumab, and MMAE via an mc-val-cit-PABC linker. The phase II trial RC48-C005 showed excellent anti-tumor activity and controllable safety of RC48 monotherapy in patients with HER2 + la/mUC after at least one systemic treatment failure [9]. RC48G001 (NCT04879329) is a phase 2 trial assessing RC48's safety, tolerance, and pharmacokinetics in HER2 + patients with la/mUC, with or without Pembro [10].

Overall, the ASCO-GU2023 Cancer Symposium has shown significant progress in the clinical trials of la/mUC. There is promising data on EV, SG and RC48, both as single and combination therapies, as summarized in Tables 1 and 2.

**Table 1:** Characteristics of ADCs for the treatment of urothelial carcinoma

ADCs	Target	mAb	Linker	Payload	DAR
EV	Nectin-4	Enfortumab	vc-PABC linker	MMAE	3.8
SG	Trop2	Sacituzumab	CL2A	SN-38	7.6
RC48	HER2	Hertuzumab	vc-PABC linker	MMAE	4

*ADCs* Antibody–drug conjugates*, CL2A* A cleavable complicated PEG8- and triazole-containing PABC-peptide-mc linker, *DAR* Drug-to-antibody ratio, *EV* Enfortumab vedotin*, HER2* Human epidermal growth factor receptor 2, *MMAE* Monomethyl auristatin E, *RC48* Disitamab vedotin, *SG* Sacituzumab govitecan, *vc-PABC* Valyl-citrullinyl-p-aminobenzyloxycarbonyl

**Table 2:** Outcomes of ADCs treatment in urothelial carcinoma from ASCO-GU 2023

Drug	Indication	Agents	Pts	ORR (%)	OS	PFS	DOR	TRAEs	NCT	References
EV	la/mUC	EV + Pembro	76	64.5	–	–	–	Skin reactions, peripheral neuropathy	NCT03288545	[1]
		EV	73	45.2	–	–	–			
	NMIBC	EV	Trials in progress						NCT05014139	[2]
SG	la/mUC	Cohort 1 SG	113	28	10.9	5.4	6.1	Neutropenia, anemia,	NCT03547973	[3]
		Cohort 2 SG	38	32	13.5	5.6	5.6	Leukopenia, fatigue		[4]
		Cohort 3 SG + Pembro	41	41	12.7	5.3	11.1	Diarrhea, febrile		[5]
		Cohort 5 SG + ZIM versus ZIM versus avelumab	Trials in progress					Neutropenia		[6]
		Cohort 6 SG versus SG + CPI versus carboplatin/GEM	Trials in progress							[7]
	mUC	SG + IPI + NIVO	6	66.6	–	8.8	9.2	Anemia, neutropenia, Pruritus, fatigue, Diarrhea, lymphopenia, arthralgia	NCT04863885	[8]
RC48	HER2 + laUC/mUC		Trials in progress						NCT04879329	[10]

*ADCs* Antibody–drug conjugates*, CPI* Checkpoint inhibitor, *DOR* Duration of response, *EV* Enfortumab vedotin, *GEM* Gemcitabine, *HER2* Human epidermal growth factor receptor 2, *IPI* Ipilimumab, *la/mUC* Locally advanced/metastatic urothelial carcinoma, *NIVO* Nivolumab, *NMIBC* Non muscle-invasive bladder cancer, *ORR*Objective response rate, *OS* Overall survival, *Pembro* Pembrolizumab, *PFS* Progression-free survival, *Pts* Patients, *RC48* Disitamab vedotin, *SG* Sacituzumab govitecan, *TRAEs* Treatment-related adverse events, *ZIM* Zimberelimab

## Data Availability

The material supporting the conclusion of this study has been included within the article.

## Abbreviations

ADC: Antibody–drug conjugate
ASCO-GU: American Society of Clinical Oncology-Genitourinary
CPI: Checkpoint inhibitor
DAR: Drug-to-antibody ratio
DOM: Domvanalimab
DOR: Duration of response
DV; RC48: Disitamab vedotin
EV: Enfortumab vedotin
FDA: Food and Drug Administration
GEM: Gemcitabine
HER2: Human epidermal growth factor receptor 2
IPI: Ipilimumab
La/mUC: Locally advanced/metastatic urothelial carcinoma
MMAE: Monomethyl auristatin E
NIVO: Nivolumab
NMIBC: Non-muscle-invasive bladder cancer
ORR: Objective response rate
OS: Overall survival
Pembro: Pembrolizumab
PFS: Progression-free survival
Pts: Patients
SG: Sacituzumab govitecan
TRAEs: Treatment-related adverse events
Vc-PABC: Valyl-citrullinyl-p-aminobenzyloxycarbonyl
ZIM: Zimberelimab
